# The involvement of E3 ubiquitin ligases in the development and progression of colorectal cancer

**DOI:** 10.1038/s41420-023-01760-z

**Published:** 2023-12-16

**Authors:** Jie Chen, Haimei Feng, Yiting Wang, Xiaoming Bai, Siqi Sheng, Huiyu Li, Mengxi Huang, Xiaoyuan Chu, Zengjie Lei

**Affiliations:** 1 Department of Medical Oncology, Jinling Hospital, Affiliated Hospital of Medical School, Nanjing University, Nanjing, Jiangsu Province China; 2 Department of Medical Oncology, Jinling Hospital, Nanjing University of Chinese Medicine, Nanjing, Jiangsu Province China; 3 Department of Medical Oncology, Jinling Hospital, Nanjing Medical university, Nanjing, Jiangsu Province China; 4 Department of Medical Oncology, Jinling Hospital, The First School of Clinical Medicine, Southern Medical University, Nanjing, Jiangsu Province China

**Keywords:** Gastrointestinal cancer, Cancer therapy

## Abstract

To date, colorectal cancer (CRC) still has limited therapeutic efficacy and poor prognosis and there is an urgent need for novel targets to improve the outcome of CRC patients. The highly conserved ubiquitination modification mediated by E3 ubiquitin ligases is an important mechanism to regulate the expression and function of tumor promoters or suppressors in CRC. In this review, we provide an overview of E3 ligases in modulating various biological processes in CRC, including proliferation, migration, stemness, metabolism, cell death, differentiation and immune response of CRC cells, emphasizing the pluripotency of E3 ubiquitin ligases. We further focus on the role of E3 ligases in regulating vital cellular signal pathways in CRC, such as Wnt/β-catenin pathway and NF-κB pathway. Additionally, considering the potential of E3 ligases as novel targets in the treatment of CRC, we discuss what aspects of E3 ligases can be utilized and exploited for efficient therapeutic strategies.

## Facts

- Colorectal cancer is the third most common cancer diagnosed worldwide, but with limited therapeutic efficacy and poor prognosis.
- E3 ligases mediated-ubiquitination regulates the expression and function of tumor promoters or suppressors in the development of CRC.
- E3 ligases play multifaceted roles in various biological processes and several vital signal pathways of CRC in a context-depending manner.

## Open questions

- Can E3 ligases be efficiently targeted to improve the treatment and prognosis of CRC?
- How can we identify the bi-functional E3 ligases in CRC and amplify their tumor suppressive effects?
- What is the mechanism that determines E3 ligases to accurately recognize and ubiquitinate different substrates in CRC cells?

## Introduction

**Fig. 1:**
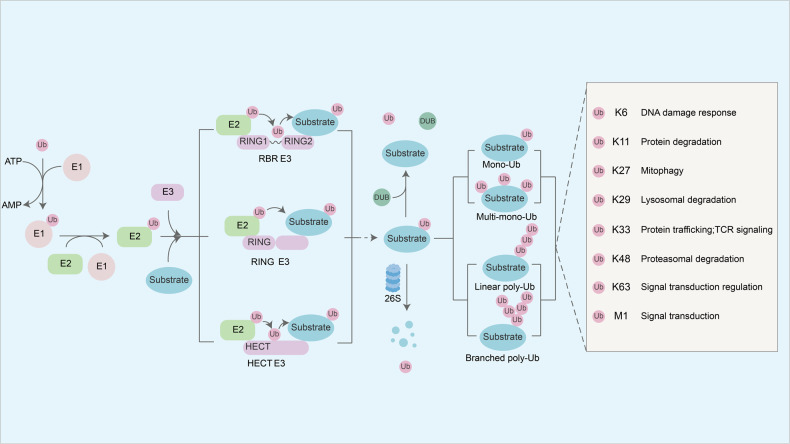
The ubiquitin-proteasome system and categories of E3 ligases as well as ubiquitination types. The ubiquitination cascade begins with the ATP-dependent activation of Ub by E1 ubiquitin-activating enzymes. After E2 ubiquitin-conjugating enzymes obtain activated-Ub, E3 ubiquitin ligases combine with Ub-E2 complex and substrate, and transfer Ub from E2 to the substrate directly or indirectly. In contrast, the deubiquitinating enzymes (DUBs) remove Ub from substrates and keep the balance of ubiquitin level of target protein together with E3 ligases to maintain cellular homeostasis. E3 ubiquitin ligases specifically recognize 7 key Lysine sites (K6, K11, K27, K33, K48 and K63) or N-terminal Methionine site (Met1) of ubiquitin and forms different chain linkages, such as mono-ubiquitination, multi-ubiquitination, linear or branched polyubiquitination chain, to exert multiple biological functions.

Protein ubiquitination is one of the predominantly post-translational modifications, and an overview of the ubiquitin-proteasome system (UPS) is provided in Fig. 1. E3 ubiquitin ligases are the key enzymes in the UPS to recognize specific substrates and covalently connect ubiquitin (Ub) to a protein substrate. E3 ligases-mediated ubiquitination modification exhibits different functions depending on the kind of ubiquitin conjugation. For instance, the most common K48-linked polyubiquitination promotes protein degradation by the 26S proteasome [1], while K63-linked polyubiquitination mainly regulates signal transduction and endocytosis [2]. The dysfunction of E3 ubiquitin ligases leads to the illegitimate degradation or abnormal accumulation of target protein, and the loss of tumor suppressive protein or the overactivation of oncogenic protein is always pathological and carcinogenic. The multifaceted impact of E3 ubiquitin ligases has been confirmed in many cancers, including breast cancer, ovarian cancer cells [3] and glioblastoma [4].

Colorectal cancer (CRC) is the third most common cancer diagnosed worldwide and remains the third leading cause of cancer mortality [5]. Despite existing treatment for CRC patients have achieved great advances, such as surgery, chemotherapy, radiotherapy, targeted therapy or immunotherapy, high recurrence rate and drug resistance constrain therapeutic efficacy and lead to poor prognosis, indicating a true desire for a more effective therapeutic modality. With growing interest of protein ubiquitination in CRC, mounting evidences have revealed that E3 ubiquitin ligases plays a pivotal and sophisticated role in the progression of CRC and suggested the promising therapeutic value of E3 ligases.

In this article, we [1] review the accumulative evidence about E3 ligases involved in modulating the proliferation, migration, stemness, metabolism, cell death and immune response of CRC cells; and [2] underline the regulatory effects of E3 ligases on classical signal pathway transduction in CRC; [3] highlight the potential of E3 ligases as molecular biomarkers and potent therapeutic targets in CRC.

## E3 ubiquitin ligases

To date, over 700 E3 ligases can be categorized into 3 main type enzymes on the basis of the structure and the process of conjugating ubiquitin to substrate: homologous to the E6-AP C terminus (HECT) domain-containing E3s, really interesting new gene (RING) finger domain-containing E3s and RING-between-RING (RBR) family E3s [6]. RING-type E3s, the largest E3 ligases family, directly transfer the ubiquitin from E2 to the substrate, while HECT-type E3s accept the E2-bound ubiquitin and then transfer it to the substrate. The smallest E3 ligases family RBR E3s contains 12 members, which have RING domain but with a transfer mechanism of ubiquitin similar to HECT-type E3s, Cullin-RING ubiquitin ligases (CRL) are multi-subunit E3 ligase complexes and consist the scaffold protein (Cullin family), the RING box protein (RBX1/2) and different substrate recognition components. For example, CRL4^DCAF1^ contains Cullin4A, RBX1, damage-specific DNA binding protein 1 (DDB1) and DDB1- and CUL4-associated factor 1 (DCAF1).

## The role of E3 ubiquitin ligases in CRC

The tumorigenesis of colorectal cancer is complicated and multi-faceted. Here we focus on the essential regulatory roles of E3 ligases in multiple biological processes closely related to CRC development, including proliferation, motility, stemness, metabolism, cell death and immune response.

### E3 ubiquitin ligases in the proliferation of CRC

Persistent proliferation of tumor cells is a hallmark of CRC, and many E3 ligases exhibits pro- or anti-proliferation effects through ubiquitinating specific proteins involved in aberrant cell cycle progression, DNA damage repair functions and other cellular responses.

As a member of tripartite motif-containing proteins (TRIM) E3 family, the oncogenic TRIM6 promotes cell proliferation through increasing the ubiquitination and degradation of TIS21, an anti-proliferative protein that arrest CRC cells in G0/G1 phase [7]. In contrast, itchy E3 ubiquitin-protein ligase (ITCH) and HECT domain and RCC1-like domain-containing protein 3 (HERC3) suppress CRC cell proliferation and lead to G0/G1 phase arrest by targeting at CDK4 and RPL23A for K48-linked ubiquitination degradation respectively [8, 9]. The oncogenic ubiquitin-protein ligase E3 component n-recognin 5 (UBR5) promotes the degradation of ECRG4 via the ubiquitin-proteasome pathway, contributing to increased S-phase cells and augmented CRC growth [10]. E3 ligase neuronal precursor cell-expressed developmentally downregulated 4 (NEDD4) is responsible for the proteasomal degradation of p21, a key negative regulator of tumor proliferation that associates with G1 arrest [11].

What’s more, E3 ligase PDZ domain containing ring finger 3 (PDZRN3) is reported to mediate K63-linked ubiquitination and degradation of TBX20, a tumor-suppressive protein that impedes Non-homologous End Joining DNA damage repair and suppresses CRC cell proliferation [12]. E3 ligase complex CRL4A^DCAF1^-mediated degradation of the ribonuclease Dicer1 promotes CRC growth [13], while β-TrCP1-induced ubiquitination and degradation of HuR, an oncoprotein that binds to proliferation-related RNA, suppresses CRC cell growth [14]. Knockdown of E3 ligase Mule exhibits anti-proliferation effect in CRC cells partially attributed to Mule-induced degradation of MIZ1, a MYC-associated protein that inhibits MYC function [15]. E3 ligase ring finger protein (RNF) 111 shows tumor-suppressive properties in tumorigenesis of CRC by mediating the proteasomal degradation of SnoN/Ski, co-repressors that block the transcription of Smad2/3 [16].

### E3 ubiquitin ligases related to the migration and metastasis of CRC

Cancer metastasis is a complicated and precisely modulated biological multi-steps process, in which epithelial–mesenchymal transition (EMT) is significant for CRC cells to acquire the invasion and migration capability.

E3 ligases F-box protein (FBXO) 11 and TRIM16 inhibit the EMT in CRC by mediating the ubiquitination and degradation of Snail, a transcription factor that represses E-cadherin expression [17, 18]. Constitutive photomorphogenic 1 (COP1) is the E3 ligase of ETV4, another transcription factor promoting CRC migration [19]. E3 ligase HERC3 induces the K27 and K48-linked ubiquitination degradation of EIF5A2, which negatively regulates EMT and suppresses CRC metastasis [20]. CRL4A^DCAF16^ complex catalyzes the mono-ubiquitination of PHGDH at K146, thereby enhances PHGDH activity to upregulate S-adenosylmethionine and promotes tumor cell migration [21]. It’s ascertained that carboxy terminus of Hsc70 interacting protein (CHIP) is the E3 ligase of autophagy-related protein 9B and myosin-9, which both contributes to CRC metastasis [22]. CHIP also mediates the ubiquitination and degradation of Mortalin-2, an oncoprotein that promotes CRC cell migration ability [23]. E3 ligase Smad ubiquitin regulatory factor (Smurf2) is responsible for the ubiquitination and degradation of RhoA, a key GTPase that contributes to metastasis in CRC [24]. TRIM65 targets at Rho GTPase activating protein 35 for proteasomal degradation, leading to increased migration-related structures in CRC cells [25].

### E3 ligases and their role in CSC stemness maintenance

The multipotent cancer stem cells (CSCs) are vital to tumorigenesis, chemotherapy resistance as well as cancer recurrence. Emerging evidences have suggested that the regulation of ubiquitination levels in CRC cells may endow cells self-renewal abilities and the E3 ligases involved in the maintenance of stemness are listed below.

E3 ligase F-box and WD repeat domain-containing (FBXW) 11 targets at tumor suppressor HIC1 for ubiquitination degradation, which contributes to the maintenance of stem-cell-like properties in CRC cells [26]. Another study found that loss of FBXW7-mediated proteasomal degradation of transcription factor ZEB2 increases CSC properties [27]. E3 ligase mouse double minute 2 (MDM2)-induced ubiquitination of p53 is enhanced by WD40 repeat domain-containing protein 35 (WDR35), leading to reduced stability of p53 and oxaliplatin resistance [28]. Overexpression of E3 ligase TRIM25 also promotes oxaliplatin resistance and the stem cell properties in CRC by blocking the K63-linked ubiquitination and degradation of EZH2 mediated by E3 ligase TNF receptor-associated factor 6 (TRAF6) [29]. TRAF6 also suppresses CSC properties by conjugating K27- and K33-linked ubiquitination to the cancer stemness marker aldehyde dehydrogenase 1 family member B1 (ALDH1B1) and decreasing its enzyme activity [30]. The autoubiquitination of E3 ligase TRIM21 at K214 and K217 is promoted by the regulator CSN6, thereby decreasing TRIM21-mediated ubiquitination and degradation of Oct1, a transcription activator of another cancer stemness marker ALDH1A1 [31]. Additionally, elevated expression of E3 ligase RANBP2-type and C3HC4-type zinc finger-containing 1 (RBCK1) is found to enhance CRC cell stemness and modulate chemosensitivity [32].

### E3 ligases in CRC cell metabolism

#### E3 ligases in glucose metabolism

Upregulated PKM2-mediated aerobic glycolysis (Warburg effect) is a hallmark of colorectal cancer cells to meet the huge energy demand for substance anabolism. E3 ligase CHIP destabilizes PKM2 via mediating its ubiquitination and degradation [33], whereas TRIM29 triggers the K48-linked ubiquitination and proteasomal degradation of PKM1 to decrease PKM1/PKM2 ratio, motivating PKM2-mediated aerobic glycolysis to become the dominate energy source of CRC cells [34]. E3 ligase FBXW7 suppresses lactate production and CRC progression by directly promoting the degradation of enolase 1 through ubiquitin-proteasome pathway [35]. FBXW7 also ubiquitinates and downregulates c-Myc via proteasomal pathway, which thereby suppresses the expression of downstream target hexokinase-2, a rate-limiting enzyme of glycolysis [36]. Moreover, Smurf2 reduce aerobic glycolysis in CRC cells through mediating ubiquitination and degradation of ChREBP, a transcription factor that can reprogram glucose metabolism [37].

#### E3 ligases in amino acid metabolism

Serine–Glycine–One-Carbon (SGOC) pathway is consisted of folate cycle and methionine cycle and supports essential metabolic processes related to the survival of CRC cells. The K95 acetylation of SHMT2, a one-carbon metabolic enzyme in folate cycle, inhibits its activity and promotes the K63-linked ubiquitination and lysosomal degradation mediated by E3 ligase TRIM21 [38]. Similarly, CRL3 E3 ligase complex-induced ubiquitination and proteasomal degradation of MAT IIα, an essential enzyme in methionine cycle, is promoted by K81 acetylation [39]. E3 ligase speckle-type POZ protein (SPOP) mediates polyubiquitination and degradation of ILF3, an oncoprotein that stabilizes the mRNA of genes involved in SGOC pathway [40]. It’s reported that E3 ligase COP1 mediates the K48-linked ubiquitination and degradation of FOXO4, which directly binds and suppresses the promoters of SGOC-related genes to attenuate SGOC metabolism [41]. E3 ligase TRAF2 decreases arginine synthesis by mediating the ubiquitination and degradation of the rate-limiting enzyme ASS1 [42].

### E3 ligases determining CRC cell fate

#### E3 ligases related to apoptosis and autophagy

Apoptosis deficiency contributes to CRC malignant phenotype. In this section, we focus on the E3 ligases involved in the complicated mechanisms of apoptosis escape and autophagy enhancement strategies in CRC cells.

E3 ligase X-linked inhibitor of apoptosis protein (XIAP) participates in the resistance against TRAIL-induced apoptosis in CRC cells through inhibiting caspase-3 activity and mediating the ubiquitination and degradation of active p17 fragment of caspase-3 [43]. In contrast, E3 ligase Mule facilitates TRAIL-induced apoptosis through inducing proteasomal degradation of Mcl-1, a member of anti-apoptotic Bcl-2 family [44]. Irradiation-induced apoptosis also involves the destabilization of Mcl-1, as irradiation decreases AKT activity and then promotes FBXW7-mediated ubiquitination of Mcl-1. Simultaneously knocking down S-phase kinase-associated protein 2 (Skp2), which mediates K63-linked ubiquitination and activation of Akt, further augments the interaction between FBXW7 and Mcl-1 as well as strengthens irradiation-induced CRC cell apoptosis [45]. FBXW7 also targets at Cyclin E for proteasomal degradation, while kinase Plk2 leads to the destabilization of FBXW7 as well as accumulation of Cyclin E and decreases apoptotic activity of CRC cells [46].

RNF152 is the E3 ligase of Bcl-xL, a highly expressed anti-apoptotic protein in CRC [47]. E3 ligase complex CRL5 is responsible for K11-linked polyubiquitination and degradation of pro-apoptotic protein Noxa in oxidative stress-induced apoptosis, which can be enhanced by peroxiredoxin 1 oligomers and contributes to inhibition of ROS-induced apoptosis [48]. Seven in absentia homolog 1 (SIAH1)-mediated proteasomal degradation of HIPK2 is attenuated by genotoxic stress, which stabilizes HIPK2 and activates apoptosis in CRC cells [49]. HECT Domain E3 Ubiquitin Protein Ligase 2 (HECTD2) mediated proteasomal degradation of EHMT2 is enhanced by propionate, leading to facilitated cell apoptosis [50]. The expression of E3 ligase HERC5 is found downregulated in CRC cells, which attenuates the ubiquitination and degradation of CtBP1, leading to suppressed apoptosis [51]. EZH2 deficiency-induced upregulation of E3 ligase ITCH enhances proteasomal degradation of the pro-survival protein c-FLIP, promoting TNFα-mediated apoptosis [52].

Autophagy is also closely related to apoptosis. CRC cell autophagy flux can be promoted by E3 ligase TRIM39, which attenuates the ubiquitination of Rab7 at Lys191, instead of augmenting its ubiquitination and degradation, and contributes to increased autophagosome-lysosome fusion and CRC progression [53]. E3 ligase TRAF6 catalyzes K63-linked polyubiquitination of the recognized marker LC3B at Lys51, contributing to autophagy activation and metastasis inhibition of CRC [54]. XIAP is the E3 ligase of p62 and inhibits p62-mediated autophagy of CRC cells [55]. E3 ligase RNF216 dampens the autophagy of CRC cells by promoting the ubiquitination and degradation of Beclin1, an indispensable regulator of autophagy [56].

#### E3 ligases related to CRC cell differentiation

Poorly differentiated CRCs are more aggressive and correlates with poor patient survival and prognosis. E3 ligase FBXW7 promotes the ubiquitination and degradation of CDX2, a transcription factor critical to intestinal differentiation in CRC progression [57]. Smurf2 mediates the poly-ubiquitination and degradation of onco-protein ID1, which inhibits cell differentiation by hindering the transcription of differentiation-related genes [58]. In addition, Smurf2 also catalyzes multiple mono-ubiquitination of Smad3 and inhibits its transcription activity, thereby downregulating ID1 and facilitating CRC cell differentiation [59]. High rate of G1-S transition is a main characteristic of poorly differentiated cells. E3 ligase Skp2 promotes CRC differentiation through mediating the ubiquitination and destabilization of p21 and p27, two CDK inhibitors that diminish G1-S transition [60]. The transcription factor RORγt is known to augment IL-17 transcription and increase Th17 cell proportion. E3 ligase ITCH targets at RORγt for ubiquitination degradation and perturbs differentiation of naive T cells, while impeding the combination of ITCH with RORγt promotes RORγt-dependent Th17 cell differentiation [61].

### E3 ligases regulating cell immune response

Overexpression of the inhibitory receptor PD-L1 on CRC cells, which contributes to silenced T cell activity and immune escape, is one of the main causes of poor response to immunotherapy of CRC patients. E3 ligase SPOP binds with the intracellular segment of PD-L1 and mediates the proteasome-dependent degradation of PD-L1, while the inactivity of SPOP contributes to immune escape in CRC patients [62]. PD-1 expressed on macrophages in CRC is ubiquitinated and degraded by casitas B lineage lymphoma (c-Cbl) to modulate the tumor microenvironment [63]. What’s more, PD-1 on tumor-infiltrating T cells is reported to be ubiquitinated and degraded by CRL3^KLHL22^ complex before its transportation to the cell surface, preventing excessive T cell suppression [64].

It’s known that activated tumor-infiltrating T cells mainly rely on glycolysis to ensure energy requirement, and the disorder of glycolytic metabolism will impair antitumor function as well as lead to immune evasion [65]. E3 ligase zinc finger protein (ZFP91) mediates K63-linked ubiquitination of the catalytic subunit of serine/threonine protein phosphatase 2A (PP2Ac) which restricts mTORC1-mediated T cell glycolytic metabolism, and facilitates the assembly of PP2A, ultimately disrupting the glycolysis and antitumor activity of T cells [66]. Different from activated T cells, activated T reg cells are commonly with lower glycolytic activity in tumor. It’s noteworthy that ZFP91 contributes to T reg cell homeostasis via inducing Beclin1 K63-linked ubiquitination, leading to inhibited mTORC1-dependent glycolysis as well as the development of immune tolerance [67].

### The pluripotency of E3 ligases

E3 ligases that are associated with a plethora of biological processes in CRC, including cell proliferation, metastasis, stemness maintenance, cell metabolism, cell fate determination and cell immune response, are listed in Table 1. It is obviously that E3 ligases shows versatility and complexity in CRC, as aberrant expressed E3 ligases simultaneously modulate various events related to tumorigenesis through recognizing different molecules and changing the activity of target proteins via mono- or poly-ubiquitination. For instance, Smurf2 negatively influences CRC progression and is identified to be the E3 ligase of RhoA, ChREBP, SIRT1, SATB1 and Smad3 [24, 37, 68–70]. E3 ligase RNF40 suppresses apoptosis by mediating H2B mono-ubiquitination of anti-apoptotic proteins genes, including Mcl-1 and BIRC5, and maintaining their expression [71]. Similarly, RNF2 inhibits CRC cell apoptosis by suppressing EGR1 expression via mediating the mono-ubiquitination of H2A at K119 of EGR1 promoter [72].

**Table 1:** The pluripotency of E3 ligases in the biological processes of CRC.

E3 ligase	Type of E3 ligase	Substrate(s)	Role in CRC	Biological processes influenced by multifunctional E3	Reference(s)
c-Cbl	RING-type E3	PD-1	tumor suppressor	immune response	[63]
CHIP	RING-type E3	ATG9B; myosin-9; Mortalin-2; PKM2;	tumor suppressor	proliferation; metastasis; aerobic glycolysis	[22, 23, 33]
COP1	RING-type E3	ETV4; FOXO4	bifunctional protein	migration; glucose metabolism; serine metabolism	[19, 41]
CRL3-KLHL22 complex	RING-type E3	PD-1	tumor suppressor	immune response	[64]
CRL3	RING-type E3	MAT IIα	tumor suppressor	proliferation; methionine metabolism	[39]
CRL4A-DCAF1 complex	RING-type E3	Dicer1	tumor promoter	proliferation; apoptosis	[13]
CRL4A-DCAF16 complex	RING-type E3	PHGDH	tumor promoter	migration	[21]
CRL5	RING-type E3	NOXA	tumor promoter	apoptosis	[48]
FBXO11	RING-type E3	Snail	tumor suppressor	migration	[18]
FBXW7	RING-type E3	CHD6; ZEB2; ENO1; c-myc; Mcl-1; Cyclin E; CDX2	tumor suppressor	proliferation; metastasis; stemness; chemoresistance; glycolysis; apoptosis; cell differentiation	[27, 35, 36, 45, 46, 57, 130]
MDM2	RING-type E3	P53	tumor promoter	proliferation; chemoresistance	[28]
PDZRN3	RING-type E3	TBX20	tumor promoter	DNA damage response	[12]
RNF2	RING-type E3	histone H2A	tumor promoter	proliferation; invasion; apoptosis	[72]
RNF6	RING-type E3	SHP-1	tumor promoter	proliferation; metastasis	[131]
RNF40	RING-type E3	histone H2B	tumor promoter	growth; apoptosis	[71]
RNF41	RING-type E3	ErbB3/ErbB4; Dvl2; ASB6	bifunctional protein	stemness; metastasis	[74, 75]
RNF111	RING-type E3	SnoN/Ski	tumor suppressor	proliferation	[16]
RNF152	RING-type E3	Bcl-xL	tumor suppressor	apoptosis	[47]
SIAH1	RING-type E3	HIPK2	tumor promoter	apoptosis	[49]
Skp2	RING-type E3	p21/p27	tumor promoter	cell differentiation	[60]
SPOP	RING-type E3	ILF3; PD-L1	tumor suppressor	growth; serine metabolism; immune response	[40, 62]
TRAF2	RING-type E3	ASS1	tumor suppressor	metastasis; arginine metabolism	[42]
TRAF6	RING-type E3	EZH2; ALDH1B1; LC3B	tumor suppressor	stemness; autophagy	[29, 30, 54]
TRIM6	RING-type E3	TIS21	tumor promoter	proliferation	[7]
TRIM16	RING-type E3	Snail	tumor suppressor	migration	[17]
TRIM21	RING-type E3	MICALL2; OCT1; TRIM21; SHMT2	tumor suppressor	growth; metastasis; stemness; serine metabolism	[31, 38, 132]
TRIM25	RING-type E3	/	tumor promoter	stemness; chemoresistance	[31]
TRIM29	RING-type E3	PKM1	tumor promoter	proliferation; metastasis; aerobic glycolysis	[34]
TRIM39	RING-type E3	/	tumor promoter	proliferation; migration; autophagy	[53]
TRIM47	RING-type E3	SMAD4	tumor promoter	proliferation; metastasis; chemoresistance	[133]
TRIM65	RING-type E3	ARHGAP35	tumor promoter	metastasis	[25]
UBE4A	RING-type E3	EPHA2	tumor suppressor	growth; invasion	[134]
XIAP	RING-type E3	OGT; p62	bifunctional protein	proliferation; invasion; autophagy	[55, 135]
ZFP91	RING-type E3	PP2Ac; BECN1	tumor promoter	immune response	[66, 67]
β-TrCP1	RING-type E3	HuR	tumor suppressor	proliferation	[14]
β-TrCP2	RING-type E3	ZNF281; HIC1	bifunctional protein	proliferation; migration; stemness	[26, 73]
RBCK1	RBR-type E3	/	tumor promoter	stemness; chemoresistance	[32]
RNF216	RBR-type E3	BECN1	tumor promoter	proliferation; migration; autophagy	[56]
HECTD2	HECT-type E3	EHMT2	tumor suppressor	growth; apoptosis	[50]
HERC3	HECT-type E3	RPL23A; EIF5A2	tumor suppressor	proliferation; metastasis	[9, 20]
HERC5	HECT-type E3	CtBP1	tumor suppressor	apoptosis	[51]
ITCH	HECT-type E3	CDK4; c-FLIP; RORγt	tumor suppressor	proliferation; apoptosis; cell differentiation	[8, 52, 61]
Mule	HECT-type E3	MIZ1; Mcl-1	bifunctional protein	proliferation; apoptosis	[15, 44]
NEDD4	HECT-type E3	p21	tumor promoter	proliferation; metastasis	[11]
Smurf2	HECT-type E3	SIRT1; SATB1; RhoA; ChREBP; Smad3	tumor suppressor	proliferation; metastasis; aerobic glycolysis; cell differentiation	[24, 37, 68–70]
UBR5	HECT-type E3	ECRG4	tumor promoter	proliferation	[10]

What’s more, instead of synergistic effects, a specific E3 ligase involved in CRC progression could be oncogenic or tumor-suppressive or have opposing functions in a context-dependent manner. For example, FBXW11 plays a dual role in tumorigenesis of CRC, which suppresses on CRC proliferation and migration through targeting at ZNF281 [73], and meanwhile contributes to stemness by downregulating HIC1 [26]. RNF41 acts as an oncogenic E3 ligase by promoting the ubiquitination and degradation of Disheveled 2 (Dvl2) in the presence of KITENIN, rather than as a suppressor of CRC through targeting at ErbB3/ErbB4 for degradation [74]. What’s more, RNF41 suppresses CRC stemness and metastasis through mediating the ubiquitination and degradation of ASB6 [75].

## E3 ligases regulating important cellular signaling pathway

### EGFR signaling pathway

**Fig. 2:**
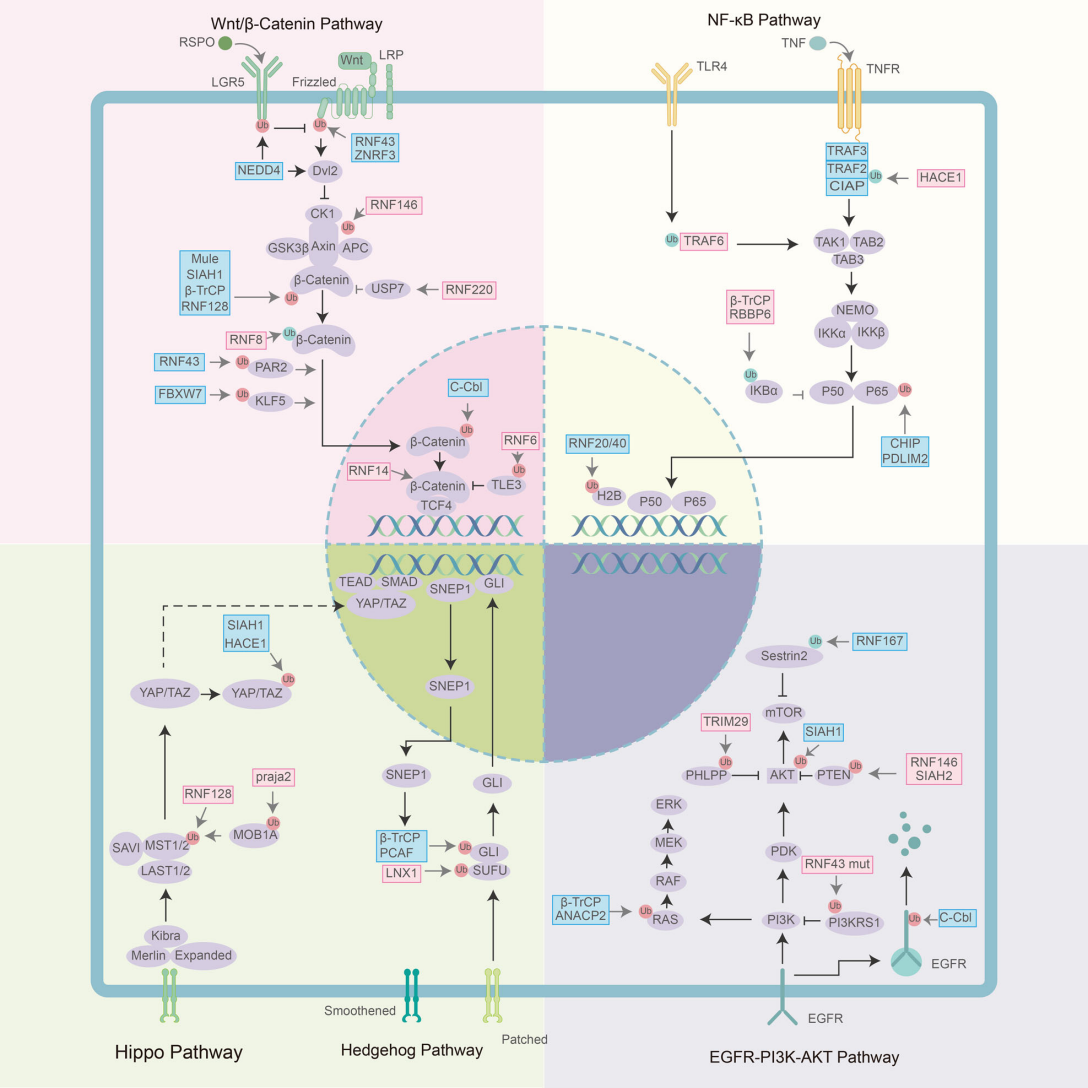
The role of E3 ligases in modulating important cellular signaling pathway in CRC, including EGFR signaling, Wnt/β-catenin signaling, NF-κB signaling, Hedgehog signaling and Hippo signaling. The ubiquitination modification marked in red destabilizes the substrate, while the ubiquitination marked in blue promotes the stability. Pink icons represent upregulated or oncogenic E3 ligases observed in CRC models. Blue icons represent downregulated or tumor-suppressive E3 ligases observed in CRC models.

The aberrant overexpression of the canonical RTK receptor EGFR is very common in CRC patients and contributes to poor prognosis. Activated EGFR initiates several phosphorylation cascades, including Ras-MEK-MAPK signaling and PI3K-AKT-mTOR signaling. Tyr1045 phosphorylation of EGFR triggers its internalization, followed by c-Cbl-mediated ubiquitination and lysosomal degradation as shown in Fig. 2, which is a common mechanism for downregulating EGFR excessive activation [76]. E3 ligase β-TrCP and anaphase-promoting complex subunit 2 (ANACP2) both suppress EGFR signaling and contribute to the destabilization of Ras in the ubiquitin–proteasome pathway [77, 78].

AKT activity is downregulated by E3 ligase SIAH1 through mediating K48-linked ubiquitination and proteasomal degradation of AKT in CRC cells [79]. In contrast, SIAH2 and RNF146 positively regulates PI3K-AKT signaling pathway by promoting the ubiquitination and degradation of PTEN, a suppressor of AKT that blocks its phosphorylation [80, 81]. AKT signaling is also negatively modulated by phosphatase PHLPP, which is ubiquitinated and degraded by TRIM29 [82]. Activated AKT directly phosphorylates and activates downstream mTORC1, whereas RNF167 mediates the K63-linked ubiquitination of Sestrin2, which inhibits mTORC1 activation by sequestering GATOR2 [83]. It’s worth noting that the mutant E3 ligase also play a role in regulating PI3K-AKT pathway, as RNF43_p.G659fs exhibits oncogenic ability through mediating the ubiquitination and degradation of PI3KRS1 and ultimately activates PI3K-AKT pathway, rather than the canonical Wnt pathway [84].

### Wnt/β-catenin pathway

Abnormal activation of Wnt signaling is widely present in CRC. The classical Wnt/β-catenin signal is initiated by the binding of Wnt ligands to a receptor complex composed of the Frizzled (FZD) receptors and the low-density lipoprotein receptors (LRPs), which is negatively regulated by E3 ligases RNF43 and its homolog zinc and ring finger protein 3 (ZNRF3) through ubiquitinating FZD receptor and inducing FZD endocytosis [85]. R-spondins (RSPO) are secreted Wnt agonists and generally neutralizes RNF43/ZNRF3 by interacting with leucine-rich repeat containing G protein-coupled receptors (LGRs), enhancing Wnt signaling under low-dose Wnt, which is attenuated by tumor-suppressive E3 ligases NEDD4 and its homologue NEDD4L through targeting LGR5 for both lysosomal and proteasomal degradation [86]. Interestingly, RSPO2 functions as a tumor suppressor by increasing the accumulation of ZNRF3 and suppressing Wnt signaling [87]. Additionally, RSPO2-augmented ZNRF3-mediated ubiquitination and degradation of FZD7 counteracts Wnt5a stimulation and inhibits noncanonical Wnt signaling [88].

As the main effector of Wnt/β-catenin signal, β-catenin is ubiquitinated and degraded by β-TrCP after phosphorylation mediated by a destruction complex consisted of Axin, APC, CK1 and GSK3β in the absence of Wnt stimulation [89]. RNF146 mediates the ubiquitination and degradation of Axin1 and positively regulates β-catenin signaling [90]. The constitutive destruction of cytosolic β-catenin is usually impeded by Dvl2 after Wnt stimulation, which can be targeted by NEDD4 and NEDD4L for ubiquitination and degradation [86]. What’s more, RNF128, c-Cbl, SIAH1 and Mule are novel E3 ligases that promote the ubiquitin-mediated degradation of β-catenin respectively in CRC [91–94]. In contrast, RNF8 conjugates K63-linked ubiquitination to β-catenin and facilitates its nuclear translocation [95]. E3 ligase FBXW7 ubiquitinates and degrades KLF5, an oncoprotein that facilitates the nuclear localization of β-catenin [96]. RNF43 also mediates the ubiquitination and degradation of protease-activated receptor 2 (PAR2) that promotes β-catenin nuclear localization [97]. Surprisingly, independent of its E3 ligase activity, RNF220 promotes the deubiquitination and stabilization of β-catenin by bridging the interaction of deubiquitinase USP7 and β-catenin [98]. Similarly, RNF14 acts as a binding partner of T-cell factor/lymphoid enhancer-binding factor (TCF/LEF) and facilitates the nuclear recruitment of β-catenin without altering H2B ubiquitination [99]. What’s more, RNF6 activates the Wnt/β-catenin pathway via accelerating the ubiquitination and proteasomal degradation of TLE3, an inhibitor of β-catenin/TCF4 complex [100].

### NF-κB pathway

In TNF-activated canonical NF-κB pathway, HECT domain and Ankyrin repeat containing E3 ubiquitin-protein ligase 1 (HACE1)-induced K63-linked ubiquitination of TRAF2 within the TNFR complex, along with cIAP1/2-dependent K63-linked ubiquitination of RIP1, both promotes the formation of TAK1/TAB2/TAB3 complex, which activates downstream IκB kinase (IKK) complex [101, 102]. E3 ligase TRAF6 is recruited after toll-like receptor 4 (TLR4) activation and mediates K63-linked ubiquitination of itself, further promoting NF-κB signal transduction [103]. NF-κB1 (p50) and RelA (p65) are ultimately liberated and act as the main effector of NF-κB pathway via transcription regulation. Under the signal activation, the inhibitor of p50/p65 dimer, IκBα, is targeted by E3 ligase RB binding protein 6 (RBBP6) and β-TrCP for ubiquitination and degradation, enabling the nuclear translocation of p50/p65 dimer [104, 105]. In contrast, E3 ligase CHIP and PDZ and LIM domain 2 (PDLIM2) promotes the ubiquitination and destabilization of p65 and inhibits NF-κB signaling [106, 107]. Downregulated RNF20/RNF40 decreases histone H2B monoubiquitylation in CRC, which recruits p65 and augments the transcription of p50/p65 dimer, consequently increasing NF-κB signaling [108].

### Hedgehog pathway

Abnormal activation of Hedgehog signaling is commonly considered essential to CRC development. E3 ligases such as P300/CBP-associated factor (PCAF) and β-TrCP participate in the regulation of Hedgehog pathway through mediating the ubiquitination and degradation of GLI proteins, the main downstream effectors of Hedgehog pathway [109, 110]. SuFu is a classical suppressor of Hedgehog pathway that prevents the nuclear accumulation of GLI. Ligand of Num-protein X 1 (LNX1) is responsible for the ubiquitination and degradation of SuFu, which is promoted by SuFu negating protein 1 (SNEP1). SNEP1 is also a target gene of GLI2, thus forming a positive feedback loop and strengthening Hedgehog signaling [111].

### Hippo pathway

The famous tumor-suppressive Hippo pathway comprises a series of phosphorylation cascade. Activated MST1/2 and SAV1 phosphorylates and activates LATS1/2 complex, which phosphorylates two oncogenic transcriptional co-activators, YAP and its orthologue TAZ, preventing their nuclear translocation and promoting their ubiquitination and degradation [112]. E3 ligase RNF128 mediates the ubiquitination and degradation of MST protein and suppresses Hippo signal [113]. Another study identified that the E3 ligase praja2 facilitates the proteasomal degradation of MOB1A, a strengthener of Hippo signal by activating LATS1/2 complex, and inhibits the Hippo signaling cascade in CRC [114]. SIAH1 directly interacts with YAP and promotes its K48-linked ubiquitination and proteasomal degradation in CRC cells [79]. HACE1 also targets YAP for degradation via proteasome pathway [115].

## The therapeutic value and application prospects of E3 ligases in CRC

In the past few years, extensive efforts have been conducted to clarify the pivotal role of E3 ligases in many biological processes and signal pathways, driving a deeper understanding of the nonnegligible roles of E3 ligases in CRC development and progression. Many E3 ligases are considered potential diagnostic markers and survival predictors of CRC patients, for instance, patients with stage II/III CRC and high-expression of an E3 ligase of c-Myc, membrane-associated guanylate kinase, WW and PDZ domain containing 3 (MAGI3), had a satisfying recurrence-free survival (~80%, 5-year) and with no necessity for further adjuvant chemotherapy [116].

### E3 ligases as novel effectors of treatment methods

Marvelous studies attempt to explore the full power of E3 ligases as novel targets of cancer therapeutics and emphasize its potential clinical significance. Researchers found the subunit of CRL complex, KLHL22, involves in the synergistic effect of 5-FU and anti-PD-1 therapy, in that 5-FU inhibits the transcription of KLHL22 and reduces the subsequent degradation of PD-1 [64]. Another study clarified that the combination of artesunate with WNT974 (a WNT inhibitor) induces the proteasomal degradation of overexpressed oncoprotein KRAS in CRC via significantly upregulating E3 ligase ANACP2 and β-TrCP [78]. Moreover, E3 ligases are the effectors of several anti-tumor active ingredients, including Scutellarein and Kurarinone, which respectively enhances cell division cycle 4 (CDC4)-mediated degradation of RAGE [117] and WDR76-mediated degradation of KRAS [118] in ubiquitin-proteasome pathway.

### E3 ligases as potential targets of cancer therapeutics

Modulating the activity of E3 ligases improves the tumor suppressive effect of current inmmuno- or chemo-therapies in CRC cells, which has great potential to be exploited therapeutically. For instance, knockout of C-cbl prevents the exhaustion of CAR-T cells and heightens the efficacy of immunotherapy [119]. MDM4/MDM2 double knockdown in CRC cells revives p53 activity and has synergistic effect with anti-tumor drugs, including cytotoxic 5-FU [120] and MEK inhibitor trametinib [121], but another study found that impaired activity of MDM2 confers irradiation resistance to CRC cells [122], suggesting that regulating E3 enzyme activity is a double-edged sword and deserves further investigations before the translation into clinical trials. Apart from silencing the expression of E3 ligase to repress the catalytic activity, a recent study defined Hakin-1, the first small molecule inhibitor targeting at the E3 ligase Hakai, blocks Hakai-dependent ubiquitination of E-cadherin in CRC cells, presenting promising therapeutic potential against CRC progression [123]. The oncogenic CRL4^DCAF4^ complex, composed of Cullin4, RBX1, DDB1 and DCAF4, mediates the ubiquitination and degradation of tumor suppressor ST7 [124]. A novel compound NSC1892 is identified to strongly disrupt the Cullin4-DDB1 interaction and cause the degradation of DDB1, thereby leading to stabilization of ST7 and significant inhibition of CRC growth [125]. Additionally, inhibiting components of CARM1-p300-c-Myc-Max (CPCM) transcriptional complex that binds to the promoter of Cullin4, also decreases the ubiquitination and degradation of ST7 [126], suggesting that it’s practical to regulate E3 ligase complex activity by targeting at its subunits.

### E3 ligases-based strategies: proteolysis targeting chimera (PROTAC) and molecular glue

Considering the fact that the 20 S proteasome inhibitors (bortezomib, carfifilzomib and ixazomib) that target ubiquitin-proteasome pathway are only satisfying in haematological malignancies and have disappointing results from clinical trials on CRC patients [127], scientists have been committed to exploring new therapeutic strategies based on ubiquitin pathway and have defined two novel techniques that targets specific oncogenic proteins based on several E3 ligases, the proteolysis targeting chimera (PROTAC) and molecular glue. PROTACs are synthetic bifunctional compounds containing a chemical linker and two ligands, with one targeting an E3 ligase and the other binding to the protein of interest (POI), artificially inducing the ubiquitination and degradation of the target. The end that recruits POI can be peptides, small molecules and antibodies depending on the aberrantly expressed proteins in CRC. Many PROTACs have entered clinical testing in breast and prostate cancer [128], and the major anti-tumor PROTACs developed in CRC are listed in Table 2. Cereblon (CRBN), the substrate receptor of CRL4, is one of the most widely used E3 ligases and can be recruited by Thalidomide and its derivatives. Von Hippel-Lindau (VHL) is another frequently targeted E3 ligase and acts as an adaptor protein of CRL2. Similarly, the molecular glue, a smaller molecule with higher bioavailability and without the requirement of a unique binding pockets in target proteins, also promotes selective protein degradation via acting as a scaffold of protein-E3 ligase interactions [129]. Overall, although it’s a highly challenging task to translate laboratory achievements into ultimately clinical efficacy, drug-induced proteolysis of target proteins based on E3 ligases-mediated ubiquitination and degradation is a promising therapeutic strategy in colorectal cancer and worth more attention.

**Table 2:** Major anti-tumor PROTACs and molecular glues found in CRC.

Name	Target	E3 ligase	Type	Reference
MDEG-541	MYC, GSPT1/2, PLK1	CRBN	PROTAC	[128]
17f	PDEδ	CRBN	PROTAC	[136]
xStAx-VHLL	β-catenin	VHL	Peptide PROTAC	[137]
TSM-1	STAT3	CRBN	PROTAC	[138]
degrader 1	NTMT1	VHL	PROTAC	[139]
ARV-825	BRD4	CRBN	PROTAC	[140]
degrader P7	SOS1	CRBN	PROTAC	[141]
A1874	BRD4	MDM2	PROTAC	[142]
BETd260/BETd246	BET	CRBN	PROTAC	[143]
8e/8j	TTK	CRBN	PROTAC	[144]
SPMI-HIF2-1	MDM2/MDMX	VHL	Stapled peptide PROTAC	[145]
brequinar-PROTAC	DHODH	VHL	PROTAC	[146]
ZNRF3*IGF1R PROTAB	IGF1R	ZNF43/ZNRF3	PROTAB	[147]
ZNRF3*HER2 PROTAB	HER2	ZNRF3	PROTAB	[147]
E7820	CAPERα	DCAF15	molecular glue	[148]
CQS	CAPERα	DCAF15	molecular glue	[148]
Indisulam	CAPERα	DCAF15	molecular glue	[148]
IMiDs	FAM83F	CRBN	molecular glue	[149]
NCT02	CCNK/CDK12	DDB1	molecular glue	[129]
FL118	DDX5	/	molecular glue	[150]

## Conclusions and future perspectives

The dynamic balance of protein is important to cellular homeostasis in colorectal cancer and E3 ubiquitin ligases are key factors mediating protein ubiquitination and degradation. The aberrant expression or regulation of E3 ligases leads to the excessive degradation or accumulation of target protein, which contributes to cell malignant transformation. In this review, we summarized the E3 ubiquitin ligases found in CRC and reveal their role in the development of CRC, including cell proliferation, migration, stemness maintenance, metabolism, cell fate determination, immune response as well as several typical signal pathways in CRC. We pointed out the pluripotency of E3 ubiquitin ligases that E3 ligases and substrates are not just biunique in CRC backgrounds. We also highlighted that it’s of great potential that E3 ubiquitin ligases become potent cancer therapeutic targets or biomarkers and it’s promising to include E3 ligase-based therapeutics in future clinical practice for the benefit of CRC patients.

## Data Availability

Data sharing is not applicable to this review as no data sets are generated and analyzed in this study.
